# Associations of *Schistosoma mansoni* Infection, Latent Tuberculosis, Host Interferon-γ Concentrations, and Praziquantel Treatment in Tanzanian Adults

**DOI:** 10.4269/ajtmh.25-0021

**Published:** 2025-11-25

**Authors:** Khanh Pham, Enock Miyaye, Maureen Ward, Crispin Mukerebe, Nsia Ulomi, Shigella Moshi, Loyce Mhango, Peter Lutonja, Danielle de Jong, Govert van Dam, Paul L. A. M. Corstjens, Daniel W. Fitzgerald, Robert N. Peck, Jennifer A. Downs, Hyasinta Jaka

**Affiliations:** ^1^ Division of Infectious Diseases, Weill Cornell Medicine, New York, New York;; ^2^ Center for Global Health, Weill Cornell Medicine, New York, New York;; ^3^ Department of Urology, Muhimbili University of Health and Allied Sciences, Dar es Salaam, Tanzania;; ^4^ Mwanza Research Centre, National Institute for Medical Research, Mwanza, Tanzania;; ^5^ Department of Cell and Chemical Biology, Leiden University Medical Center, Leiden, The Netherlands;; ^6^ Department of Parasitology, Leiden University Medical Center, Leiden, The Netherlands;; ^7^ Mwanza Intervention Trials Unit, National Institute for Medical Research, Mwanza, Tanzania;; ^8^ Department of Medicine, Weill Bugando School of Medicine, Mwanza, Tanzania;; ^9^ Department of Internal Medicine, Catholic University of Health and Allied Sciences-Bugando, Mwanza, Tanzania

## Abstract

Latent tuberculosis infection (LTBI) and *Schistosoma mansoni* are common in Africa, and helminth-induced immunomodulation may affect LTBI detection. This study aimed to assess whether *S. mansoni* infection affects LTBI detection by the QuantiFERON-TB Gold Plus (QFT-Plus) assay and alters serum interferon-γ (IFN-γ) concentrations in response to *Mycobacterium tuberculosis* (*Mtb*) antigens at baseline and after 1 year, during which participants with *S. mansoni* infection received praziquantel treatment. At baseline, 65 individuals with schistosome infection had lower average IFN-γ concentrations in TB1-stimulated QFT-Plus supernatants compared with 83 uninfected individuals (10.4 versus 51.9 pg/mL, *P* = 0.038). Although not statistically significant, QFT-Plus test positivity rate was unexpectedly slightly higher among adults with schistosome infection at baseline (26.2% versus 18.1%, *P* = 0.24). The incidence over 12 months was higher posttreatment in participants initially infected with *S. mansoni* compared with those uninfected (13.9% [*n* = 5/36] versus 4.2% [*n* = 2/48], *P* = 0.13). By 12 months, IFN-γ concentrations were comparable between the two groups (53.8 versus 33.5 pg/mL, respectively, *P* = 0.56). Individuals who cleared *S. mansoni* infection experienced a nearly 12-fold increase in IFN-γ levels relative to those who remained uninfected, although this difference did not reach statistical significance (*P* = 0.17). In conclusion, baseline *S. mansoni* infection was associated with suppressed IFN-γ responses to *Mtb* antigens, suggesting helminth-induced immune dampening. Praziquantel treatment may partially restore TB-specific immune responses and facilitate LTBI detection. These findings highlight the potential role of *S. mansoni* as a critical cofactor affecting LTBI diagnosis in schistosomiasis-endemic regions.

## INTRODUCTION

Latent tuberculosis infection (LTBI) and *Schistosoma mansoni* are both highly prevalent in Africa, with coinfections occurring frequently.^1^^–^^4^ Tuberculosis (TB) is a leading cause of global mortality.^5^ Detecting LTBI offers an opportunity for preventive treatment to reduce TB-related morbidity and mortality.^6^

Latent TB infection screening is typically conducted using interferon-γ release assays (IGRAs), such as the QuantiFERON-TB Gold Plus (QFT-Plus; Qiagen, Venlo, The Netherlands), or tuberculin skin testing.^7^ These tests are not perfect, and because they rely on host immune responses to *Mycobacterium tuberculosis* (*Mtb*) antigens, test sensitivity may decrease in immunocompromised individuals or those with altered T cell-mediated immune and/or interferon-γ (IFN-γ) responses, resulting in a false-negative or indeterminate IGRA result.^6^^,^^7^ Additionally, according to one cohort study, approximately one third of individuals who developed active TB were previously IGRA negative.^8^ Both innate and adaptive immune responses are essential to control *Mtb* as a latent infection, preventing progression to active disease.^9^ Interferon-γ activates innate immune cells and is critical for host survival in the context of LTBI.^9^

Schistosomes suppress host T helper 1 (Th1) responses, including IFN-γ secretion, while promoting T helper 2 (Th2) responses.^10^^,^^11^ Prior research has documented downregulation of the IFN-γ signaling pathway and DNA hypermethylation of the Th1 pathway that persists at least 6 months after praziquantel treatment in asymptomatic children exposed to TB who had *Schistosoma hematobium* infection compared with uninfected controls.^10^ Based on these findings, we hypothesized that schistosome infections would affect immune response to LTBI and therefore, QFT-Plus test performance. Specifically, we predicted that people with *S. mansoni* would have lower serum IFN-γ concentrations in response to *Mtb* antigen stimulation at baseline. We also hypothesized that previously negative or indeterminate QFT-Plus results would turn positive as LTBI and IFN-γ responses both increase after the restoration of host immune function after praziquantel treatment and the elimination of schistosome infection.

Therefore, in this prospective cohort study, we determined the effects of *S. mansoni* infection and praziquantel treatment on QFT-Plus test positivity and the detection of LTBI over 12 months. We also quantified baseline and 12-month QFT-Plus TB1 supernatant IFN-γ concentrations to quantify the effects of schistosome infection on the IFN-γ response to *Mtb* antigens.^12^

## MATERIALS AND METHODS

### Baseline participant selection.

**Figure 1.**
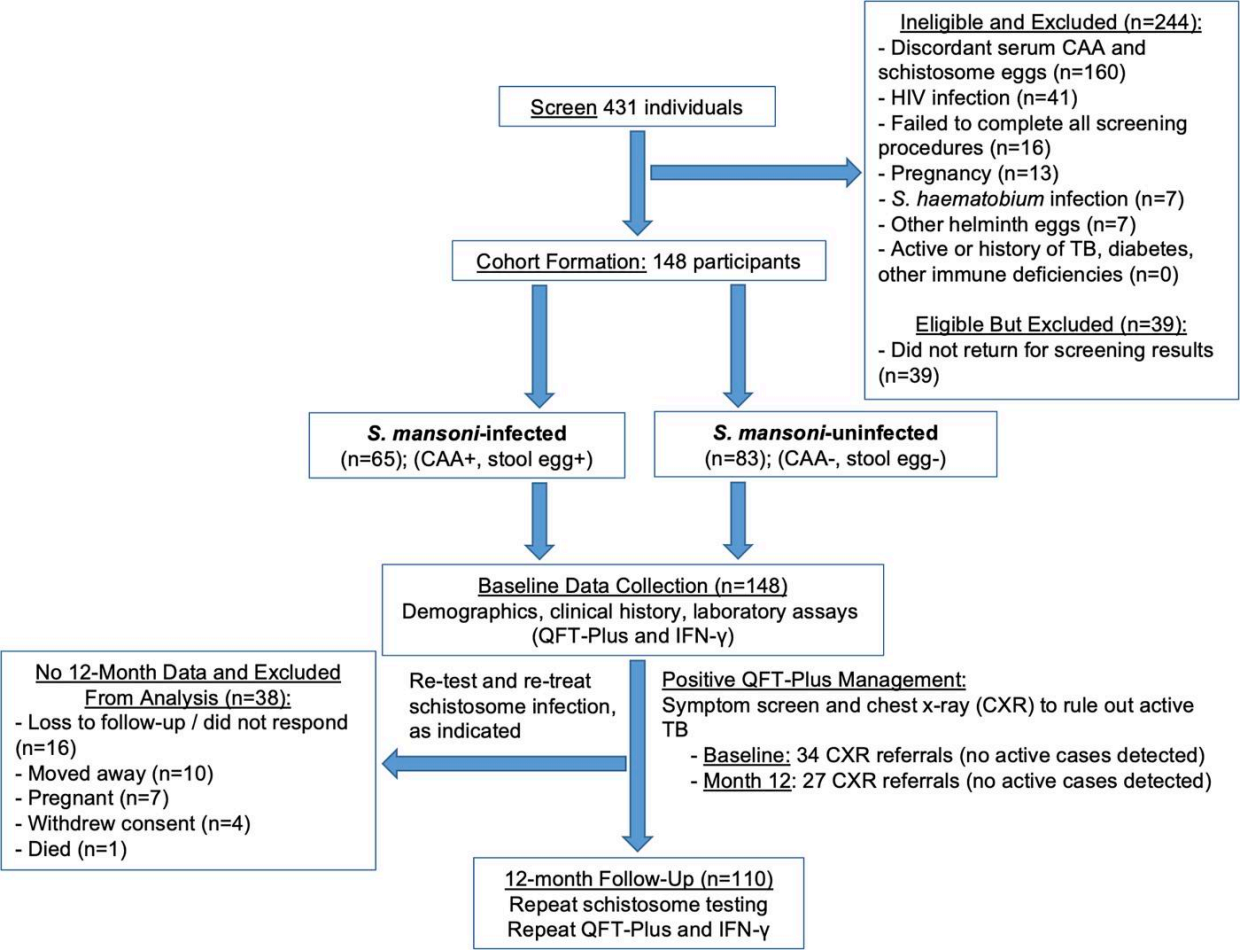
Flowchart of study procedures. CAA = circulating anodic antigen; CXR = chest X-ray; IFN-γ = interferon-γ; QFT-Plus = QuantiFERON-TB Gold Plus; TB = tuberculosis.

Following previously published study procedures,^13^ we recruited 148 Tanzanian adults ages 18–50 years old with no reported comorbidities living with and without *S. mansoni* infection in northwest Tanzania (Kisesa and Kayenze) from 2022 to 2024 (Figure 1), where the prevalence of *S. mansoni* is approximately 30% and where we have established protocols to optimize community sensitization, recruitment, and follow-up. We powered the study to detect differences in QFT-Plus test positivity and IFN-γ responses as the primary outcomes. Given the variability of LTBI prevalence,^1^ we predicted that 15% of schistosome-infected individuals versus 36% of schistosome-uninfected individuals would have a positive QFT-Plus result, with a corresponding 25% lower IFN-γ response (SD of 15%) in those with schistosome infection.^14^ Therefore, enrolling 148 individuals between these two groups would give us 83% and >99%, respectively, to detect these differences.

These 148 individuals provided written informed consent for the collection of blood, urine, and stool to test for HIV, schistosome infection, and LTBI with measurement of IFN-γ in the QFT-Plus TB1 supernatant. People with HIV were excluded from the study because of the known immunosuppressive effects of HIV.

### Twelve-month participant selection.

All participants from the baseline cohort were invited to return for collection of blood for repeat HIV counseling and testing and blood, urine, and stool collection for repeat schistosome and LTBI testing.

### *Schistosoma mansoni* infection diagnosis and treatment.

We defined *S. mansoni* infection as a serum circulating anodic antigen (CAA) ≥30 pg/mL, at least one stool ovum with *S. mansoni* morphology, and a negative urine microscopy for *S. hematobium*.^13^^,^^15^ Those found with *S. mansoni* infection were provided praziquantel under direct observation of study personnel. Every participant enrolled was offered follow-up repeat testing and treatment of schistosome infection at months 2, 4, 6, 9, and 12.

### Latent TB infection diagnosis and treatment.

Latent TB infection was defined as having a positive QFT-Plus test (conventional cutoff ≤8.0 IU/mL for “nil” negative control plus ≥0.35 IU/mL for either “TB1 minus nil” or “TB2 minus nil” antigen plus ≥25% of nil according to the package insert) plus a negative TB symptom screen and chest X-ray. All participants who tested QFT-Plus positive or indeterminate underwent a TB symptom screen and chest imaging according to the Tanzanian national TB guidelines.^16^ The country’s national guidelines do not currently recommend LTBI treatment in HIV-uninfected adults.^16^

### Laboratory testing.

#### Schistosome testing.

In the field, five slides were prepared from one stool sample and examined microscopically by a trained parasitologist using the Kato–Katz method. The use of five slides from a single stool sample has a sensitivity equivalent to multiple stool specimens examined on different days.^17^ Ten milliliters of urine were filtered, and the filter was examined microscopically for *S. hematobium* eggs. Serum CAA was quantified using the serum anodic antigen 20-*µ*L (SCAA20) assay test, indicating active *Schistosoma* infection for samples with CAA concentrations above the assay threshold of 30 pg/mL.^13^

#### QFT-Plus testing.

Trained laboratory staff obtained 4 mL of blood for IGRA testing in the field, which consisted of collecting 1 mL directly into each of the four tubes provided in the QFT-Plus Blood Collection Tubes kit (Qiagen, Venlo, The Netherlands): negative control (“nil”), positive mitogen control (“mitogen”), and *Mtb*-specific antigen (“TB1” and “TB2”) tubes. QuantiFERON-TB Gold Plus quantification was performed on fresh blood samples at the National Institute for Medical Research (NIMR) laboratory in Mwanza. We followed the manufacturer’s package insert and maintained the blood collection tubes between 17°C and 25°C at the time of blood filling; then, we transferred them to a 37°C ± 1°C incubator as soon as possible (required within 16 hours of collection). Because our study sites are 30–45 minutes away from NIMR, we were able to efficiently transport collected samples from the field to the laboratory, ensuring that they met the manufacturer’s recommendations for sample collection and processing. Once the specimens were received at NIMR, the entire QFT-Plus test was immediately conducted over 2 consecutive days, which included incubation of the specimens in the QFT-Plus Blood Collection Tubes upright at 37°C ± 1°C for 16–24 hours.

#### IFN-γ quantification.

After the above incubation period and before the ELISA was performed, the remaining supernatants of TB1 samples were frozen and shipped to New York by World Courier (London, England). At the Weill Cornell Global Health Laboratories, quantitation of IFN-γ was performed using a Luminex immunoassay (Invitrogen, Waltham, MA). The assay was run on a Magpix system and analyzed with Luminex xPONENT software (build 31.871.0; Luminex, Austin, TX). Samples were run in singlet and undiluted as recommended by the manufacturer. Every plate run had standard curve samples run in duplicate. Samples found out of the standard range were repeated at a higher or lower dilution.

#### Fasting finger-stick glucose testing.

At enrollment and follow-up visits, we measured random blood glucose by finger stick. We followed Tanzanian national guidelines to interpret and manage blood glucose measurements, including how to determine diabetes status and management.^16^ Finger-stick testing was performed because of the known effect of diabetes on host susceptibility to infectious diseases, including TB.

## STATISTICAL ANALYSES

Data were collected using printed case report forms (CRFs), which were available in English, translated into Kiswahili, and previously used in our studies. Real-time quality control was conducted by a different study team member during the same study visit to verify the accuracy and completeness of the data recorded by another team member. The CRFs were then double entered into Research Electronic Data Capture (REDCap), a PHP-based system available to Weill Cornell researchers that securely stores collected data on a regularly backed-up server.^18^ Biweekly quality control checks were also performed on the entered data to identify any errors, which were then reviewed by the study team.

Data were analyzed in Stata IC v. 17 (StataCorp, College Station, TX). We used descriptive statistics with means and SDs for continuous variables and frequencies and percentages for categorical variables. Both univariable and multivariable regression models were used, the latter adjusting for age and sex given differences between schistosome infection groups for these two covariates. Incident LTBI was also assessed and suggested by a baseline QFT-Plus test that was negative or indeterminate but converted to positive at the 12-month follow-up. A χ^2^ test was used to assess differences in incident LTBI, whereas a paired *t*-test was used to quantify changes in IFN-γ. We also used sex-by-schistosome-infection interaction terms in the multivariable models to assess for effect modification by sex on QFT-Plus and IFN-γ results. *P* <0.05 was considered significant.

## RESULTS

We enrolled 148 individuals (Figure 1), of whom 110 attended their follow-up visit at month 12. Throughout the study, no cases of active TB were identified, and as a result, no treatment referrals were made per Tanzanian national guidelines for management of LTBI in adults without HIV infection.

### Pretreatment baseline evaluation.

At baseline, participants with (*n* = 65) and without *S. mansoni* infection shared similar demographic and clinical characteristics (Table 1) except that the *S. mansoni* uninfected group tended to be older (mean age of 34.5 versus 31.5 years old among individuals with *S. mansoni* infection, *P* = 0.06) and had a higher proportion of women (51.8% versus 33.9% women among those with *S. mansoni* infection, *P* = 0.03).

**Table 1:** Baseline demographic and clinical characteristics of 148 adults with and without *Schistosoma mansoni* infection

Characteristic, *n* (%) or Mean (SD)	Schistosome Infected, *n* = 65	Schistosome Uninfected, *n* = 83
Age, years	31.5 (8.2)	34.5 (10.0)
Sex (female)*	22 (33.9%)	43 (51.8%)
Marital status		
Single	12 (18.5%)	15 (18.1%)
Married	43 (66.2%)	50 (60.2%)
Other	10 (15.4%)	18 (21.7%)
Years attended school	7.7 (2.4)	8.5 (2.3)
Ever received schistosome treatment in the past	18 (27.7%)	17 (20.5%)
Active tobacco use	14 (21.5%)	9 (10.8%)
Active alcohol use	13 (20.0%)	13 (15.7%)
Did not eat lunch or dinner because not enough food in last month	15 (23.1%)	18 (21.7%)
Fruit, servings per week	5.8 (6.3)	6.9 (8.2)
Vegetables, servings per week	7.0 (8.9)	8.0 (7.3)
Fasting glucose, mmol/L	4.9 (0.7)	5.1 (1.0)
Glycemic status (based on fasting glucose)		
No diabetes	39 (88.6%)	49 (76.6%)
Prediabetes	4 (9.1%)	12 (18.8%)
Diabetes	1 (2.3%)	3 (4.7%)

* *P* <0.05.

There was no significant difference in baseline QFT-Plus test positivity between individuals with and without *S. mansoni* infection. However, those with schistosome infection had nearly fivefold lower IFN-γ concentrations in TB1 supernatants compared with those without infection (10.4 versus 51.9 pg/mL, *P* = 0.038), even after adjusting for age and sex (Table 2).

**Table 2:** QuantiFERON-TB Gold Plus results of 148 adults with and without *Schistosoma mansoni* infection at baseline and 12 months

Characteristic, *n* (%) or Mean (SD)	Schistosome Infected (*n* = 65)	Schistosome Uninfected (*n* = 83)	Coefficient [95% CI], Unadjusted *P*-Value
Baseline QFT-Plus Results			
LTBI status by QFT-Plus results at baseline			
Negative	48 (73.9%)	67 (80.7%)	*P* = 0.32
Positive	17 (26.2%)	15 (18.1%)	*P* = 0.24
Indeterminate	0 (0%)	1 (1.2%)	–
Interferon-γ (pg/mL) in TB1 supernatant	10.4 (26.8)	51.9 (155.6)	−41.6 [−80.8 to −2.3], *P* = 0.038*

Characteristic, *n* (%) or Mean (SD)	Schistosome Infected (*n* = 48)	Schistosome Uninfected (*n* = 62)	Coefficient [95% CI], Unadjusted *P*-Value
Month 12 QFT-Plus results			
LTBI status by QFT-Plus results at month 12			
Negative	34 (70.8%)	47 (75.8%)	*P* = 0.64
Positive	14 (29.2%)	15 (24.2%)	*P* = 0.64
Indeterminate	0 (0%)	0 (0%)	–
Interferon-γ (pg/mL) in TB1 supernatant	53.8 (195.7)	33.5 (145.4)	20.4 [−49.0 to 89.7], *P* = 0.56

LTBI = latent tuberculosis infection; QFT-Plus = QuantiFERON-TB Gold Plus.

* *P* <0.05 in the multivariable regression model adjusting for age and sex.

Recognizing the importance of diabetes on TB pathogenesis, further analysis adjusting for diabetes status did not change the baseline or follow-up associations. We also did not see any evidence for effect modification by sex in the relationship between schistosome infection and QFT-Plus or IFN-γ results.

### Posttreatment 1-year evaluation.

Of the 148 individuals originally enrolled in the study, 38 participants were excluded from the analysis because of missing 12-month data. Reasons for loss to follow-up included nonresponse or inability to contact (*n* = 16), relocation (*n* = 10), pregnancy (*n* = 7), withdrawal of consent (*n* = 4), and death (*n* = 1).

Among 110 individuals with both baseline and 12-month data, 26 of 48 (54.1%) of those with *S. mansoni* infection at baseline were found to have schistosome infection at follow-up despite observed praziquantel therapy, often at several times during follow-up. Participants were analyzed based on baseline schistosome infection status. Mean CAA levels decreased from 8,730.6 pg/mL among the 65 individuals with baseline schistosome infection to 508.2 pg/mL among the 48 individuals who were seen at follow-up.

At 12 months, there were no significant differences between groups in any QFT-Plus parameters. However, individuals with baseline *S. mansoni* infection showed a trend toward a higher rate of QFT-Plus test conversion compared with those without infection. Among those who tested QFT-Plus test negative at baseline, the rate of QFT-Plus conversion was 13.9% (*n* = 5/36) in the *S. mansoni*-infected group versus 4.2% (*n* = 2/48) in the uninfected group (*P* = 0.13 by χ^2^ test) (Supplemental Table 1).

By 12 months, IFN-γ concentrations were similar between individuals with and without baseline *S. mansoni* infection (53.8 versus 33.5 pg/mL, *P* = 0.56). Interestingly, participants who cleared *S. mansoni* infection over the study period (*n* = 20) exhibited an almost 12-fold increase in IFN-γ levels compared with those who remained uninfected (*n* = 40), although this difference was not statistically significant (*P* = 0.17) (Supplemental Table 2).

## DISCUSSION

To the best of our knowledge, we present the first data that *S. mansoni* infection is associated with suppressed IFN-γ responses to TB antigens, consistent with helminth-induced immune modulation. Our findings also suggest that praziquantel treatment may partially restore TB-specific immune responsiveness and influence the detection of LTBI over time.

No prior studies have specifically examined the relationship between *S. mansoni* infection and QFT-Plus test positivity in individuals without HIV. In line with our findings that *S. mansoni* infection is associated with a dampened immune response to TB antigens, other studies have reported that *S. mansoni* coinfection is associated with worse outcomes in active TB, including poor treatment outcomes and increased mortality.^19^^,^^20^ Here, we documented lower IFN-γ concentrations in response to *Mtb* antigens among those with *S. mansoni* infection. Further, after intensive monitoring and treatment, participants who had baseline schistosome infection had a tendency toward greater newly positive QFT-Plus results and no longer had lower IFN-γ concentrations. This raises the possibility that active schistosome infection may be associated with impaired performance of QFT-Plus testing, potentially because of a reduction in the IFN-γ response to *Mtb*. Alternately, individuals with schistosome infection may be at increased susceptibility to LTBI acquisition or persistence. Although not statistically significant, individuals with baseline *S. mansoni* infection had both higher rates of QFT-Plus test positivity at baseline (26.2% versus 19.3%) and had higher rates of new QFT-Plus test positivity at 12 months (13.9% versus 4.2%).

Similar findings have been reported in other studies examining IFN-γ responses, including one that linked schistosome infection with lower IFN-γ signaling and lower TB-specific IFN-γ production by CD4+ T cells in children exposed to TB.^11^ Additionally, a meta-analysis indicated a lower pooled estimate of IFN-γ levels among individuals with schistosome infection, although the difference did not reach statistical significance.^21^ Our data support the conclusion that *S. mansoni* infection significantly modulates host IFN-γ responses in the setting of LTBI exposure. Specifically, *S. mansoni*-infected individuals had nearly fivefold lower IFN-γ concentrations at baseline, which rose over the course of 12 months—after praziquantel treatment—to levels comparable with those in uninfected participants. Because many participants still had schistosome infection but at lower intensity after frequent treatments, our data suggest that treatment of schistosome infection may improve the host response to TB and potentially, other infections.

Our study has several limitations. First, we assumed that LTBI would be evenly distributed between those with and without *S. mansoni* infection. However, individuals with baseline schistosome infection had higher rates of LTBI at baseline as well as more incident LTBI over the follow-up period as measured by QFT-Plus. These findings suggest that LTBI may have been more prevalent in the *S. mansoni*-infected group, potentially because of unmeasured sociodemographic or environmental factors. Second, recurrent *S. mansoni* infections during the study period complicated interpretation and may have attenuated our ability to detect differences in immune outcomes. Additionally, loss to follow-up occurred in 11% of study participants, largely because of the high mobility of individuals in fishing communities. This reduced the sample size at follow-up and may be mitigated in future studies by enrolling less transient populations. Third, the study follow-up period was limited to 12 months. A longer observation window, especially in the context of effective and sustained schistosome clearance, may have yielded more definitive insights into IGRA dynamics and LTBI progression. This is particularly important given the uncertain duration of schistosome-induced immune modulation, even after praziquantel treatment.^11^ Finally, although we relied on serum CAA and stool microscopy to assess infection status, serological testing could have provided complementary information, especially in detecting past or low-intensity infections. However, in endemic settings like ours, serology is likely to remain positive because of prior exposure, limiting its utility in distinguishing active from resolved infections. Despite these limitations, key strengths of this study include its rigorous design, use of the most advanced IGRA assay currently available, and direct quantification of IFN-γ responses in TB1-stimulated supernatants, offering a detailed view of host immune function in the context of helminth–TB coexposure.

## CONCLUSION

In conclusion, baseline *S. mansoni* infection was associated with suppressed IFN-γ responses to TB antigens, consistent with helminth-induced immune modulation. Praziquantel treatment appeared to partially restore TB-specific immune responsiveness and may influence subsequent LTBI detection. These findings underscore the potential role of *S. mansoni* as a key cofactor in shaping host immune responses and affecting the performance of IGRA-based diagnostics. Further research is needed to determine whether untreated schistosome infection increases the risk of LTBI acquisition and impairs diagnostic accuracy—questions with important clinical and public health implications in coendemic regions.

## Supplemental Materials

10.4269/ajtmh.25-0021 Supplemental Materials

## Data Availability

Deidentified data may be available upon request to qualified researchers who meet the criteria for access to confidential data. Interested researchers may contact Nao Haba (nah7023@med.cornell.edu) at Weill Cornell Medicine.
