# Supplementary material for: Associations of Schistosoma mansoni Infection, Latent Tuberculosis, Host Interferon-γ Concentrations, and Praziquantel Treatment in Tanzanian Adults
Source: Am J Trop Med Hyg. 2025 Nov 25;114(2):247–52. doi: 10.4269/ajtmh.25-0021 (PMC12874967; doi:10.4269/ajtmh.25-0021)
Supplement: Supplemental Materials [file tpmd250021.SD1.pdf]

Supplemental Table 1: Results of Baseline and Endline QFT-Plus Results in People With Baseline *S. mansoni* (Panel A) and Those Without Baseline Schistosome Infection (Panel B).

<u>Panel A. People With Baseline *S. mansoni* (n=48) Who Had Both Baseline and Endline QFT-Plus Results</u>

		**QFT Results Baseline**		
		Positive	Negative	Indeterminate
**QFT Results Endline**	Positive	9 (18.8%)	5 (10.4%)	0
	Negative	3 (6.3%)	31 (64.6%)	0
	Indeterminate	0	0	0

<u>Panel B. People Without Baseline *S. mansoni* (n=62) Who Had Both Baseline and Endline QFT-Plus Results</u>

		**QFT Results Baseline**		
		Positive	Negative	Indeterminate
**QFT Results Endline**	Positive	11 (17.7%)	2 (3.2%)	1 (1.6%)
	Negative	2 (3.2%)	46 (74.2%)	0
	Indeterminate	0	0	0

Supplemental Table 2: Interferon-gamma Concentrations Become Similar Between Individuals Who Cleared Infection Versus Those Who Remained Uninfected After 12 Months

	**Change Interferon-gamma Concentrations After 12 Months**		
	Cleared Infection (n=20) Baseline→12-Month (Difference)	Remained Uninfected (n=40) Baseline→12-Month (Difference)	Difference in Difference; p-value
Interferon-gamma (pg/mL) in TB1 supernatant	8.0→97.0 pg/mL (+89.0)	37.1→42.7 pg/mL (+5.6)	-83.4; p=0.17
